# XBP1u Is Involved in C2C12 Myoblast Differentiation *via* Accelerated Proteasomal Degradation of Id3

**DOI:** 10.3389/fphys.2022.796190

**Published:** 2022-01-27

**Authors:** Satoko Hayashi, Shotaro Sakata, Shotaro Kawamura, Yukako Tokutake, Shinichi Yonekura

**Affiliations:** ^1^Graduate School of Medicine, Science and Technology, Shinshu University, Nagano, Japan; ^2^Graduate School of Science and Technology, Shinshu University, Nagano, Japan; ^3^Department of Biomolecular Innovation, Institute for Biomedical Sciences, Shinshu University, Nagano, Japan

**Keywords:** skeletal muscle differentiation, cell cycle exit, unfolded protein response, Id3, cyclin-dependent kinase inhibitor

## Abstract

Myoblast differentiation is an ordered multistep process that includes withdrawal from the cell cycle, elongation, and fusion to form multinucleated myotubes. Id3, a member of the Id family, plays a crucial role in cell cycle exit and differentiation. However, in muscle cells after differentiation induction, the detailed mechanisms that diminish Id3 function and cause the cells to withdraw from the cell cycle are unknown. Induction of myoblast differentiation resulted in decreased expression of Id3 and increased expression of XBP1u, and XBP1u accelerated proteasomal degradation of Id3 in C2C12 cells. The expression levels of the cyclin-dependent kinase inhibitors p21, p27, and p57 were not increased after differentiation induction of XBP1-knockdown C2C12 cells. Moreover, knockdown of Id3 rescued myogenic differentiation of XBP1-knockdown C2C12 cells. Taken together, these findings provide evidence that XBP1u regulates cell cycle exit after myogenic differentiation induction through interactions with Id3. To the best of our knowledge, this is the first report of the involvement of XBP1u in myoblast differentiation. These results indicate that XBP1u may act as a “regulator” of myoblast differentiation under various physiological conditions.

## Introduction

Myoblast differentiation is an ordered multistep process that includes withdrawal from the cell cycle and the expression of key myogenic factors leading to fusion into multinucleated myotubes (Stockdale, 1992). Progression through the cell cycle phases is dependent on consecutive activation and inhibition of phosphoproteins by cyclin-dependent kinases (CDKs) complexed with associated activatory cyclins (Harashima et al., 2013). Cyclin-dependent kinase inhibitors (CKIs) are negative cell cycle regulators (Vidal and Koff, 2000). Based on their sequence homology and specificity of action, CKIs are divided into two distinct families as follows: INK4 and Cip/Kip (Sherr and Roberts, 1999). Cip/Kip members, i.e., p21^ Cip1^ (p21), p27^ Kip1^ (p27), and p57^ Kip2^ (p57) share a conserved N-terminal domain that mediates binding to cyclins and inhibit a broader spectrum of cyclin–CDK complexes (Harper et al., 1993; Polyak et al., 1994; Lee et al., 1995). CKIs inhibit CDK activity, and contribute to the cell cycle exit (Tane et al., 2014). In muscle cell differentiation, the CDK-cyclin complex is downregulated, while the expression of p21 is increased (Fujio et al., 1999; Liu et al., 2017). In mice lacking p21, the differentiation of myoblasts is decreased and cell proliferation is increased (Hawke et al., 2003; Chinzei et al., 2015). During myogenesis, p27 is upregulated to withdraw from the cell cycle (Zabludoff et al., 1998) and to promote differentiation (Leshem et al., 2000). Mice lacking both p21 and p57 fail to form myotubes, displaying increased proliferation and apoptotic rates of myoblasts (Zhang et al., 1999).

Inhibitor of DNA-binding (Id) proteins are helix-loop-helix (HLH) transcription factors that play crucial roles in cell cycle exit and differentiation (Ying et al., 2003; Ciarapica et al., 2009). In mammals, there are four Id protein isoforms: Id1–4. Id1, Id2, and Id3 can inhibit myogenic differentiation, while Id4 functions as corepressors of other HLH transcription factors (Atherton et al., 1996; Melnikova and Christy, 1996; AlSudais et al., 2018). Id3 is the most highly expressed Id family member in myoblasts but expression is decreased after differentiation (Atherton et al., 1996). Knockdown of Id3 is reported to enhance myogenic differentiation (AlSudais et al., 2018). In C2C12 mouse muscle progenitor cells overexpressing *Id3*, withdrawal from the cell cycle after differentiation induction is delayed and differentiation is inhibited (Melnikova and Christy, 1996). However, in muscle cells after differentiation induction, the mechanism underlying diminished Id3 function that causes the cell to withdraw from the cell cycle is unknown.

Skeletal muscle differentiation and formation occurs along with a significant level of cellular stress (Nakanishi et al., 2005; Jahnke et al., 2009; Baechler et al., 2019). The unfolded protein response (UPR), which is induced by stress to the endoplasmic reticulum (ER), is necessary for the differentiation of various cell types, including myoblasts (Iwakoshi et al., 2003; Sha et al., 2009; Tohmonda et al., 2011; Tsuchiya et al., 2017). Three pathways are involved in the ER stress response: PERK (protein kinase R-like ER kinase), ATF-6 (activating transcription factor 6), and IRE1α (inositol-requiring enzyme 1 α) (Schröder, 2008). Upon sensing the accumulation of unfolded proteins, IRE1α cleaves unspliced X-box-binding protein 1 (XBP1u) mRNA and removes a 26-nucleotide-long intron, resulting in the production of spliced XBP1 (XBP1s) (Yoshida et al., 2001). The majority of the described functions of XBP1 are ascribed to XBP1s. XBP1s, a transcriptional factor, regulates the transcription of specific genes, depending on the cell type, to control cellular functions, including ER homeostasis, lipid metabolism, and cell differentiation (Park et al., 2021). Indeed, our previous study demonstrated that XBP1s regulates the early stages of myogenesis (Tokutake et al., 2019). XBP1s are involved in apoptosis and autophagy occurring in the first 24 h after differentiation induction. Furthermore, the expression of cyclin-dependent kinase 5 (CDK5), intimately involved in skeletal muscle development (Lazaro et al., 1997) is regulated by XBP1s. However, XBP1u consists of a hydrophobic stretch and lacks the transcription activation domain. XBP1u contains a degradation domain and can bind XBP1s. Therefore, XBP1u controls the termination of UPR responses by mediating the proteasomal degradation of XBP1s (Yoshida et al., 2006). However, relatively few studies have investigated the role of XBP1u in cellular differentiation.

Therefore, the aim of this study was to investigate the potential role of XBP1u in Id3-mediated cell exit and inhibition of C2C12 myogenic differentiation. The results showed that Id3 expression was decreased, while XBP1u expression was increased after induction of muscle differentiation and XBP1u accelerated the degradation of Id3 *via* the proteasomes of C2C12 cells. Knockdown of Id3 rescued myogenic differentiation of XBP1-knockdown C2C12 cells. These finding suggest that XBP1u plays an important role in switching from the undifferentiated to differentiated state by targeting Id3 for degradation.

## Materials and Methods

### Reagents

Dulbecco’s modified Eagle medium (DMEM) was purchased from Invitrogen (Grand Island, NY, United States). Fetal bovine serum (FBS) was purchased from EQUITECH-BIO (Cotton Gin Lane, TX, United States). Horse serum was obtained from Thermo Scientific (Waltham, MA, United States). Precast, 4–20% Mini-PROTEAN TGX gels and Polyvinylidene fluoride (PVDF) membranes were obtained from Bio-Rad (Hercules, CA, United States). MG132, Cycloheximide (CHX), Id3 siRNA, and non-targeting control siRNA were purchased from Sigma-Aldrich (Saint Louis, MO, United States). All other compounds were purchased from Nacalai Tesque (Kyoto, Japan).

### Antibodies

For immunoblotting analysis, the following antibodies were used: anti-Myosin 4 (MF20) monoclonal antibody (Thermo Scientific), anti-Flag (catalog number: PM020) rabbit polyclonal antibody (MBL, Nagoya, Japan), anti-XBP1 rabbit polyclonal antibody (catalog number: sc-7160), anti-Id3 (catalog number: sc-56712), anti-p21 (catalog number: sc-6246), anti-cyclin D1 (catalog number: sc-8396) mouse monoclonal antibody (Santa Cruz Biotechnology), anti-α-tubulin (catalog number: PM054), anti-mouse or anti-rabbit IgG HRP-linked whole Ab (GE Healthcare, Chicago, IL, United States).

### Plasmids

The XBP1u expression plasmid was generated using standard DNA techniques. Mouse cDNA encoding Id3 (GenBank accession number NM_008321) was amplified from total RNA of skeletal muscle tissue by RT-PCR using a sense primer (5′-TCCCTCTCTATCTCTACTCTCCAAC-3′) and an antisense primer (5′-AGTCCCAGGGTCCCAAGC-3′). Flag-Id3 was produced by cloning into a pFLAG-CMV expression vector (Sigma-Aldrich). The nucleotide sequences of PCR products and construct were verified by sequencing. The sequence was analyzed using the basic local alignment search tool (BLAST). The pcDNA3.1(-) plasmid encoding XBP1u was a kind gift from Dr. Ann-Hwee Lee, Ph.D. (Regeneron Pharmaceuticals, Inc., Tarrytown, NY, United States).

### Cell Culture

C2C12 mouse myoblast cell line (DS Pharma Biomedical, Osaka, Japan) and previously generated XBP1-knockdown cell lines were cultured as described previously (Tokutake et al., 2019). Briefly, the cells were cultured in Dulbecco’s modified Eagle’s medium (DMEM) supplemented with 10% fetal bovine serum under an atmosphere of 5% CO_2_/95% air at 37°C. Primary mouse myoblasts were isolated from 4 week-old C57bl/6J mice (Japan SLC, Hamamatsu, Japan). Undifferentiated myoblasts were maintained in growth medium consisting of DMEM supplemented with 20% fetal bovine serum and 2 ng/mL basic fibroblast growth factor (FUJIFILM Wako Pure Chemical Corporation, Osaka, Japan). To induce differentiation into myotubes, cells were shifted to DMEM supplemented with 2% horse serum.

To assess degradation of Id3 by XBP1u, C2C12 cells were co-transfected with p cDNA3.1(-)-XBP1u and a Flag-Id3 construct using Lipofectamine 2000 reagent (Invitrogen Corporation, Carlsbad, CA, United States) in accordance with the manufacturer’s protocol. At 24 h post transfection, the proteasome inhibitor MG132 (20 μM) was added to the culture and the cells were incubated for 6 h.

For the proteosome experiment, at 24 h post transfection, the cell cultures were harvested, and replated in 9-cm tissue culture dishes. Then, the cells were treated with cycloheximide (CHX) (100 μg/ml) with or without MG132 (20 μM), and incubated for the indicated times.

For the knockdown experiment, XBP1-knockdown cells or primary mouse myoblasts were seeded in growth medium at a confluence of 40–50% in the wells of a 6-well plate and incubated overnight at 37°C. The next day, the cells were transfected with either small interfering RNA (siRNA) against Id3 or control siRNA using Lipofectamine 2000 reagent in accordance with the manufacturer’s protocol. For induction of myogenic differentiation, at 6 h post transfection, the cells were cultured in differentiation medium.

### RNA Extraction and Quantitative Real-Time PCR

Total RNA was isolated from C2C12 cells, XBP1-knockdown cells or primary mouse myoblasts using the TRIzol reagent (Invitrogen) following the manufacturer’s instructions. The concentration of total isolated RNA was determined by optical density measurements at 260 nm and its purity was measured at a wavelength ratio of 260/280 nm (1.85–2.0 was the acceptable value range) using a spectrophotometer (NanoDrop One Spectrophotometer, Thermo Scientific). The cDNA was synthesized from total RNA using a qPCR RT Master Mix with gDNA Remover (TOYOBO, Osaka, Japan). Quantitative real-time PCR was performed using SYBR Premix Ex Taq TM II (TaKaRa Bio Inc., Shiga, Japan). Relative expression was normalized to TATA-binding protein (*Tbp)* or Glyceraldehyde-3-phosphate dehydrogenase (*Gapdh*) gene expression. The following primers were used: *Tbp* F: 5′-cattctcaaactctgaccactgcac-3′, R: 5′-CAGCCAAGATTCACGGTAGATACAA-3′; *Gapdh* F: 5′-TTGTGATGGGTGTGAACCACGAG-3′, R: 5′-CATGAG CCCTTCCACAATGCCAA-3′; *Xbp1s* F: 5′-TGAGAACCAGGA GTTAAGAACACG-3′, R: 5′-CCTGCACCTGCTGCGGAC-3′; *Xbp1u* F: 5′-AGACTATGTGCACCTCTGCA-3′, R: 5′-ACA GGGTCCAACTTGTCCAG-3′; *Id3* F: 5′-ACATCTTCCCAT GGACTCTG-3′, R: 5′-TAGGTCCTTCTGGGTAGACC-3′; *Myogenin* F: 5′-TACGTCCATCGTGGACAGCAT-3′, R: 5′- TCAGCTAAATTCCCTCGCTGG-3′; *Myh1* F: 5′-AGAGCC AAGAGGAAACTGGAGG-3′, R: 5′- CTCGTCCTCAATCTTG CTCTGC-3′; *p21/Cdkn1a* F: 5′-GCAGACCAGCCTGACAGAT TT-3′, R: 5′-GAGAGGGCAGGCAGCGTAT-3′; *p27/Cdkn1b* F: 5′-TCAAACGTGAGAGTGTCTAACG-3′, R: 5′-CCGGGC CGAAGAGATTTCTG-3′; *p57/Cdkn1c* F: 5′-GTAGCAGGAAC CGGAGATGG-3′, R: 5′-TTTACACCTTGGGACCAGCG-3′; *Ccnd1* F: 5′-CGGATGAGAACAAGCAGACC-3′, R: 5′-GCGGT AGCAGGAGAGGAAGT-3′; Relative transcript expression was calculated by the 2^–*ddCt*^ method (Pfaffl, 2001) and represented as relative values to control or 0 h.

### Co-immunoprecipitaion and Immunoblotting

The cells were harvested and lysed in RIPA lysis buffer [50 mM Tris–HCl (pH 7.4) containing 1% NP-40, 0.25% sodium deoxycholate, 0.1% SDS, 150 mm NaCl, 1 mM EDTA, and 1 × protease inhibitor cocktail (Nacalai Tesque)] to prepare protein extracts. For coimmunoprecipitation, a Pierce Crosslink magnetic IP and Co-IP kit (Thermo Scientific) was used to capture Id3-Flag-binding cellular proteins by co-immunoprecipitation (co-IP) according to the manufacturer’s instruction. The cell extracts or co-IP samples were size-fractionated using SDS-PAGE and protein bands were subsequently transferred to PVDF membranes. Membranes were incubated with primary followed by secondary antibodies in blocking buffer. Labeled proteins were visualized using the ECL Prime Western Blotting Detection Reagent kit (GE Healthcare); images were captured using an Image Quant LAS 500 (GE Healthcare) and analyzed with ImageJ software from the NIH^1^.

### EdU Proliferation Assay

At 0, 12, 24, and 48 h after differentiation stimuli, cell proliferation was detected using incorporation of 5-*ethynyl*-2′-deoxyuridine (EdU) with the Click-iT EdU Cell Proliferation Assay Kit (Invitrogen). Briefly, cells were incubated with 10 μM EdU for 1 h before fixation, permeabilization, and EdU staining, which were carried out according to the kit’s protocol. The cells were incubated in a DAPI solution for 5 min. Fluorescence photographs were taken on EVOS^®^ FL Auto (Life Technologies; Carlsbad, CA, United States). Quantification of proliferation nuclei was accomplished by performing counts for DAPI and EdU. Nuclei were counted manually using digital photography and Adobe Photoshop software.

### Immunocytochemistry and Myotube Quantification

Immunocytochemistry was performed as described previously (Tokutake et al., 2015). Briefly, cells were washed with PBS, fixed with 4% paraformaldehyde in PBS, and blocked with 10% goat serum in PBS with 0.01% Triton-X 100. Then, the cells were incubated with mouse anti-Myosin 4 (MF20) monoclonal antibody (Thermo Scientific) (1:50) for 2 h at room temperature. The cells were then incubated with Alexa-Fluor^®^ 488-conjugated goat anti-mouse antibody (Thermo Scientific) for 1 h. The cell nuclei were stained with DAPI (Thermo Scientific) and observed under the EVOS^®^ FL Auto (Thermo Scientific). The differentiation potential of the myoblasts, known as the fusion index, was evaluated as a percentage of the number of nuclei contained within MF20-positive myotubes per total number of nuclei. At least 500 nuclei from ten random fields were counted for group (*n* = 3).

### Statistical Analysis

All data are presented as the mean ± standard error of the mean (SEM) of at least three independent experiments. Comparisons between two samples were conducted using the Student’s *t*-test or Mann–Whitney *U* test, while comparisons of multiple groups were performed using analysis of variance followed by the *post hoc* Tukey–Kramer’s honestly significant difference test. A probability (*p*) value of < 0.05 was considered statistically significant.

## Results

### XBP1u Expression Was Increased After Differentiation Induction of C2C12 Cells

**FIGURE 1:**
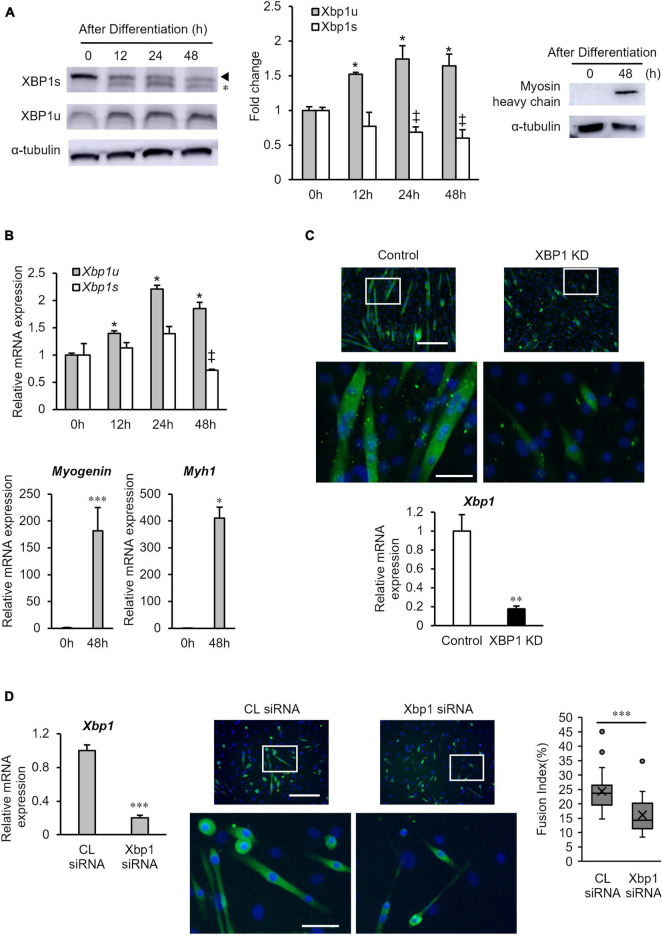
XBP1u expression is increased after differentiation induction. **(A)** XBP1s and XBP1u protein expression levels in C2C12 cells during differentiation. C2C12 cells were treated with the proteasome inhibitor MG132 (20 μM) for 2 h prior to lysate preparation. Protein levels of each XBP1 isoform and were detected by western blot analysis. *Non-specific band. Left panel: Representative images of three independent experiments are shown. Middle panel: Quantification of XBP1 isoform expression obtained from three independent experiments and normalized to α-tublin as represented in the bar graph. The results are presented as the mean ± SEM (*n* = 3). Student’s *t*-test. *‡*p* < 0.05 vs. 0 h of each group. Right panel: Protein level of myosin heavy-chain (MHC) was detected *via* western blot analysis. Representative images of three independent experiments are shown. **(B)** Relative expression of XBP1s, XBP1u, Myogenin, and Myh1 mRNA were determined by RT-qPCR. The results are presented as the mean ± SEM (*n* = 3). Student’s *t*-test. *‡*p* < 0.05, ^***^p < 0.001 vs. 0 h of each group. **(C)** Upper panel: Image comparison of the control and XBP1-knockdown myotubes. Five days after differentiation, cells were immunostained using an anti-myosin heavy-chain antibody (green). Nuclei were stained with DAPI (blue). Scale bar = 400 μm. The images below show larger magnification views of boxed regions. Scale bar = 50 μm. Lower panel: The mRNA level of *Xbp1* was determined by RT-qPCR and normalized to *Gapdh*. The results are presented as the mean ± SEM of three independent determinations. Student’s *t*-test. ^**^p < 0.01. KD indicates “knockdown.” **(D)** Primary mouse myoblasts were transfected with XBP1 siRNA or control siRNA. Left panel: The mRNA level of *Xbp1* was determined by RT-qPCR and normalized to *Gapdh*. The results are presented as the mean ± SEM of three independent determinations. Student’s *t*-test. *p < 0.05 vs. the control group. Middle panel: Three days after differentiation, cells were immunostained using an anti-muscle heavy-chain antibody (green). Nuclei were stained with DAPI (blue). Representative fluorescent images of the primary mouse myoblasts treated with control siRNA or XBP1 siRNA in differentiation medium for 72 h. Scale bar = 400 μm. The images below show larger magnification views of boxed regions. Scale bar = 50 μm. Right panel: The fusion index was calculated. The data are representative of four independent experiments. Ten views were analyzed for each experiment. Mann–Whitney *U* test. ^***^p < 0.001 vs. the control group.

First, the protein expression levels of XBP1s and XBP1u in differentiated cells were measured by western blot analysis. As shown in Figure 1A, XBP1u expression was significantly increased at 12 h after differentiation induction, whereas XBP1s expression was significantly decreased at 24 h after differentiation induction and remained low. The mRNA expression levels of *XBP1s* and *XBP1u* were also assessed by quantitative real-time polymerase chain reaction (RT-qPCR). As shown in Figure 1B, mRNA expression of *XBP1u* was significantly increased after differentiation induction, whereas *XBP1s* mRNA expression was decreased. We confirmed that protein expression of myosin heavy-chain (MHC) and mRNA expression of *Myogenin* and *Myh1* increased 48 h after differentiation (Figures 1A,B). Consistent with our previous report, myotube formation in previously generated XBP1-knockdown C2C12 cell lines that stably expressed XBP1 shRNA (XBP1- knockdown cells), was inhibited 5 days post differentiation induction (Figure 1C). Our previous study has also showed that the expression of myogenesis related genes (*MyoD, Myogenin, Mrf4*, and *Mef2c*) was significantly repressed with differentiation in XBP1-knockdown cells (Tokutake et al., 2019). To confirm the role of XBP1 in other type of myoblast, XBP1 was knocked down in primary-cultured mouse myoblasts by siRNA. RT-qPCR data confirmed that *Xbp1* expression in the knockdown cells was significantly lower compared with that in control cells. XBP1 silencing impaired myogenic differentiation of primary-cultured mouse myoblasts. The fusion index (average number of myonuclei/MyHC + cells) of knockdown cells was significantly lower than that in control cells (Figure 1D).

### Id3 Expression Was Maintained at a Higher Level After Differentiation Induction of XBP1- Knockdown Cells

**FIGURE 2:**
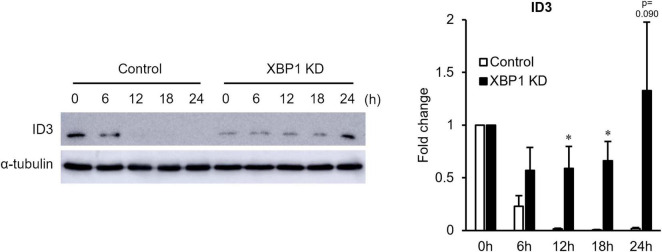
ID3 protein expression level in XBP1-knockdown cells during early differentiation. Control or XBP1-knockdown cells were induced to differentiate and treated with the proteasome inhibitor MG132 (20 μM) for 6 h before lysate preparation. Id3 and α-tubulin (internal control) protein levels were detected by western blot analysis. Left panel: Representative images of four independent experiments are shown. Right panel: Quantification of Id3 expression obtained from four independent experiments and normalized to α-tublin as represented in the bar graph. The results are presented as the mean ± SEM (*n* = 4). Student’s *t*-test. *p < 0.05. “KD” indicates “knockdown.”

Since Id3 is an important factor of early stage myogenic differentiation, Id3 protein expression was monitored in the early differentiation stage. As shown in Figure 2, XBP1 depletion maintained high Id3 expression after differentiation induction, although Id3 expression rapidly diminished in control cells. These results suggest that maintenance of Id3 expression is associated with differentiation inhibition of XBP1-knockdown cells.

### XBP1u Accelerated Proteasomal Degradation of Id3 in C2C12 Cells

**FIGURE 3:**
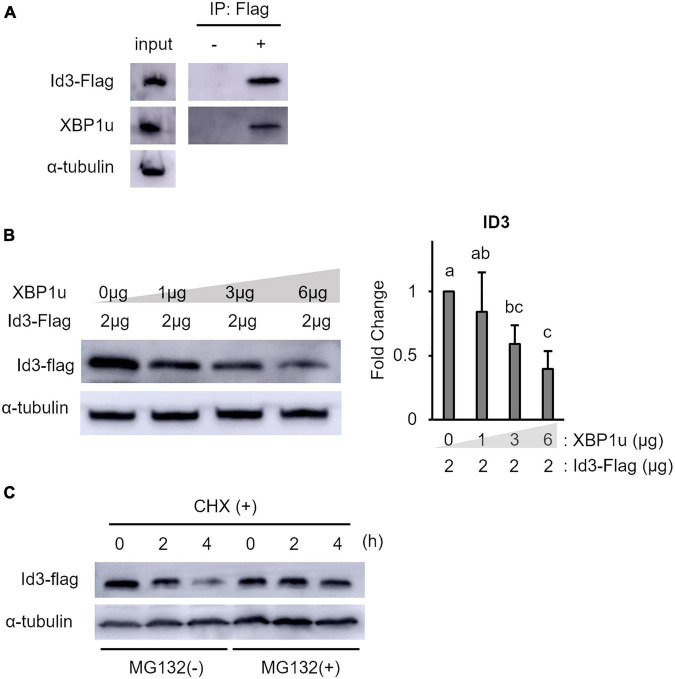
XBP1u proteins were co-immunoprecipitated with Id3 and ectopic XBP1u decreased the level of ectopic Id3 in C2C12 cells. **(A)** Co-immunoprecipitation of ectopic XBP1u with ectopic Flag-Id3 from cell lysates. C2C12 cells were co-transfected with pcDNA3.1-XBP1u and Flag-Id3 expression plasmids, then treated with MG132 (20 μM) for 6 h before lysate preparation. The lysates were immunoprecipitated with anti-Flag antibodies and, as a negative control, with rabbit IgG. The immunoprecipitates were subjected to western blot analysis with antibodies against Id3 or FLAG. Aliquots of the lysates (input) were similarly subjected to western blot analysis. Representative images of three independent experiments are shown. **(B)** Ectopic XBP1u decreased the levels of ectopic Id3 proteins in C2C12 cells in a dose-dependent manner. C2C12 cells in 3.5-cm dishes were transfected with 2 μg of Flag-Id3 expression plasmids and, if indicated, with pcDNA3.1-XBP1u. After 36 h of incubation, the cells were lysed in radioimmunoprecipitation assay buffer, and the levels of Id3 and α-tubulin (internal control) were determined by western blot analysis. Left panel: Representative images of three independent experiments are shown. Right panel: Quantification of Id3 expression obtained from three independent experiments and normalized to α-tublin as represented in the bar graph. The results are presented as the mean ± SEM (*n* = 3), Tukey–Kramer test. Means with different letters are significantly different, *p* < 0.05. **(C)** Xbp1u accelerated proteosomal degradation of ectopic Id3-Flag in C2C12 cells. C2C12 cell were co-transfected with pcDNA3.1-XBP1u and Flag-Id3 expression plasmids. After 24 h, the cultures were digested with trypsin/EDTA, pooled, replated, and incubated for a further 24 h. Then, the cells were treated with CHX (100 μg/ml) with or without MG132 (20 μM) and incubated for the indicated times. The cells were lysed and the levels of Id3-Flag and α-tubulin (internal control) were determined by western blot analysis. Representative images of three independent experiments are shown.

XBP1u is a negative mediator of XBP1s, ATF6, and Foxo1 by targeting these molecules for proteasomal degradation (Yoshida et al., 2006, 2009; Zhao et al., 2013). Therefore, to determine whether XBP1u interacts with Id3, co-expression of XBP1u and Id3 was induced in C2C12 cells. Co-immunoprecipitation analysis revealed that XBP1u was physically bound to Id3 in transfected cells (Figure 3A). As shown in Figure 3B, there was an ectopic XBP1u dose-dependent decrease in Id3 expression. Degradation of Id3 is reported to occur through the ubiquitin-proteasome pathway (Bounpheng et al., 1999). Western blot analysis revealed that in C2C12 cells, in which protein synthesis was blocked by CHX, the degradation of Id3 was inhibited by MG132, an agent that blocks protein degradation by proteasomes (Figure 3C). These results indicate that XBP1u accelerated proteasomal degradation of Id3 in C2C12 cells.

### XBP1-Knockdown Exhibited Abnormal Proliferation and CDK Inhibitor Expression After Differentiation Induction

**FIGURE 4:**
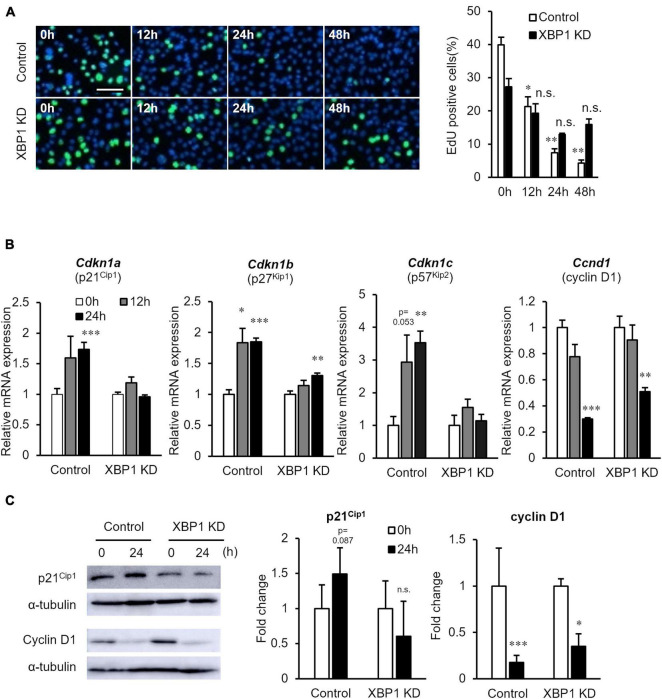
XBP1-knockdown promoted abnormal proliferation and CDK inhibitor expression after differentiation induction. **(A)** EdU staining of control or XBP1-knockdown cells. Control or XBP1-knockdown cells were induced to differentiate and then incubated in EdU and 4′,6-diamidino-2-phenylindole (DAPI) solution for the indicated times. Left panel: Images showing EdU incorporation (green) and DAPI (blue). Right panel: Quantification of the percentage of EdU-positive nuclei. The results are presented as the mean ± SEM of three independent experiments. Welch’s *t*-test. *p < 0.05, ^**^p < 0.01 vs. 0 h of each group. “n.s.” indicates not significant. Scale bar = 100 μm. **(B)** The mRNA expression levels of *Cdkn1a*, *Cdkn1b*, *Cdkn1c*, and *Ccnd1* during differentiation induction, as determined by RT-qPCR and normalized to *Gapdh*. The results are presented as the mean ± SEM of four independent experiments. Student’s *t*-test. *p < 0.05, ^**^p < 0.01, ^***^p < 0.001 vs. 0 h of each group. **(C)** p21 and cyclin D1 protein expression levels after differentiation induction of control or XBP1-knockdown cells. Control or XBP1-knockdown cells were induced to differentiate and cell lysates were prepared at the indicated times. The expression levels of p21, cyclin D1, and α-tubulin (internal control) were determined by western blot analysis. Left panel: Representative images of three independent experiments are shown. Right panel: Quantification of p21 or cyclin D1 expression of three independent experiments and normalized to α-tublin and represented in the bar graph. The results are presented as the mean ± SEM of three independent experiments. “KD” indicates “knockdown.” Student’s *t-*test. *p < 0.05, ^***^p < 0.001 vs. 0 h of each group.

Next, the proliferation of XBP1-knockdown cells after differentiation induction was examined. The EdU incorporation assay showed that XBP1-knockdown cells maintained the ability to proliferate after differentiation induction, while proliferation of control cells was significantly reduced (Figure 4A). In order to further investigate the mechanism of XBP1-knockdown to alter the cell cycle after differentiation induction, we identified key cell cycle regulatory genes. The mRNA expression levels of the CDK inhibitors *p21*, *p27*, and *p57* were significantly increased after differentiation induction of control cells (Figure 4B). On the other hand, the expressions levels were unchanged in XBP1- knockdown cells. The mRNA expression level of cyclin D1 was decreased after differentiation induction in both control and XBP1-knockdown cells (Figure 4B). Western blot analysis was performed to measure p21 and cyclin D1 protein expression levels to confirm that XBP1-knockdown alters the cell cycle after differentiation induction. As shown in Figure 4C, XBP1-knockdown altered p21 protein expression after differentiation induction, but not cyclin D1 expression. These results indicate that XBP1-knockdown altered the expression of the CDK inhibitor.

### Loss of Id3 Rescues XBP1-Knockdown-Mediated Inhibition of Myogenic Differentiation

**FIGURE 5:**
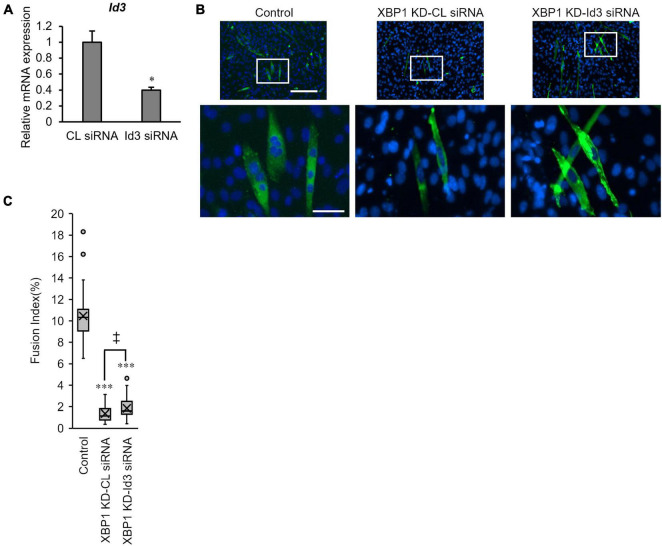
Loss of Id3 rescues XBP1- knockdown -mediated inhibition of myogenic differentiation. **(A)** C2C12 cells were transfected with Id3 siRNA or control siRNA. The mRNA level of *Id3* was determined by RT-qPCR and normalized to *Gapdh*. The results are presented as the mean ± SEM of three independent determinations. Student’s *t*-test. *p < 0.05 vs. the control group. **(B)** XBP1- knockdown cells were transfected with Id3 siRNA or control siRNA prior to differentiation induction. Cells were immunostained with anti-MHC antibody (green) and DAPI (blue) on day 3 post differentiation. Scale bars = 400 μm. The images below show larger magnification views of boxed regions. Scale bar = 50 μm. **(C)** The fusion index was calculated. KD indicates “knockdown.” The data are representative of four independent experiments. Ten views were analyzed for each experiment. Mann–Whitney *U* test. ^***^p < 0.001 vs. the control group, ‡*p* < 0.05 CL siRNA vs. Id3 siRNA.

Finally, the effect of Id3 silencing on myogenic differentiation in XBP1-knockdown cells was investigated. XBP1-knockdown cells were treated with Id3 siRNA prior to differentiation induction. RT-qPCR confirmed the knockdown of *Id3* (Figure 5A). The fusion index of XBP1-knockdown cells was partially rescued by knockdown of Id3 (Figure 5B).

## Discussion

The intracellular mechanisms underlying myoblast differentiation and cell cycle withdrawal remain unclear. It is well known that Id3 is involved in both myoblast proliferation and differentiation. The results of this study demonstrated that XBP1u, a UPR-related molecule, plays a role in Id3-mediated myoblast differentiation.

XBP1 is a major regulator of the UPR and mediates adaptation to ER stress. XBP1s is a key transcriptional factor that regulates the transcription of genes involved in the UPR (Frakes and Dillin, 2017). Additionally, XBP1s contributes to the differentiation of various cell types (Iwakoshi et al., 2003; Sha et al., 2009; Tohmonda et al., 2011; Tsuchiya et al., 2017). Indeed, our previous study demonstrated that XBP1-knockdown remarkably suppressed C2C12 myoblast differentiation and the expression of CDK5 (cyclin-dependent kinase 5), which is associated with myogenic cell differentiation and patterning and regulated by XBP1s (Philpott et al., 1997; Tokutake et al., 2019). In this study, however, expression of XBP1u, but not XBP1s, was increased after differentiation induction of C2C12 cells (Figure 1A). This is the first report of increased XBP1u expression after differentiation induction. However, further studies are needed to identify the mechanisms underlying the regulation of XBP1u expression after differentiation induction.

XBP1u has no transcriptional activity (Calfon et al., 2002) and undergoes rapid proteasomal degradation (Tirosh et al., 2006). Although relatively short-lived, XBP1u has a degradation domain and acts as a negative regulator of the UPR by targeting XBP1s and activates ATF6 for degradation (Yoshida et al., 2006, 2009). Therefore, XBP1u is thought to act as a regulator involved in fine-tuning of the UPR. Moreover, XBP1u affects autophagy by interacting with the transcription factor FOXO1 (Zhao et al., 2013). In addition, XBP1u physically bound to ID3 accelerated the proteasomal degradation of Id3 (Figure 3). ID3, which is expressed at a higher level after differentiation induction of XBP1-knockdown cells (Figure 2), prevents skeletal muscle differentiation (Melnikova and Christy, 1996). Taken together, these results suggest that XBP1u degrades Id3 after differentiation induction of C2C12 myoblasts. Moreover, the results suggest that XBP1u plays an unexpectedly important role as a regulator, at least in response to differentiation induction.

Cell cycle arrest is critical for muscle differentiation. Exit from the cell cycle is accomplished by the down-regulation of cyclins, with the exception of cyclin D3 (Skapek et al., 1995), and induction of the CDKIs p21, p57, and p27, which inhibit a wide range of CDKs essential for cell cycle progression (Guo et al., 1995; Halevy et al., 1995; Sherr and Roberts, 1999). In the present study, abnormal proliferation and the expression levels of CDKIs p21, p27, and p57 were unchanged after differentiation induction of XBP1-knockdown cells (Figure 4). Also, XBP1-knockdown myoblasts exhibited abnormal proliferation (Supplementary Figure 1). A recent study indicated that XBP1u downregulated p21 expression (Huang et al., 2017). Moreover, Id3 is a novel regulator of CDKIs, which could lead to decreased expression of p21 accompanied by proliferation (Mueller et al., 2002). Silencing of Id3 primarily attenuated p21 and p27 expression (Sharma et al., 2012), although Id3 appears to be involved in the control of the steady-state level of p27 at the G1/S boundary (Chassot et al., 2007). Further, Id3 potently repressed expression of the p57 (Lee et al., 2011). Therefore, the abnormal proliferation of XBP1-knockdown cells may be due to the maintenance of Id3 expression.

Finally, the loss of Id3 rescued XBP1-knockdown -mediated inhibition of myogenic differentiation (Figure 5). Therefore, abnormal cell cycling after differentiation induction may be a factor in abnormal differentiation of XBP1-knockdown cells. However, only partial phenotype rescue was observed, as XBP1s is also involved in muscle differentiation. The results of our previous study implied that XBP1s is necessary for myogenic cell adaptation and viability upon differentiation induction (Tokutake et al., 2019). Also, cell cycle exit during osteogenic differentiation of mesenchymal stem cells is reportedly mediated by Xbp1s-induced upregulation of p21 and p27 (Zhang et al., 2020). Considering that Xbp1s is involved in the expression of genes related to the cell cycle and cell adaptation as a transcription factor, and that XBP1u regulates the expression of other protein, including XBP1s and Id3 *via* its degradation domain, it is expected that XBP1s and XBP1u regulate the muscle differentiation process in a coordinated and complex manner. Further studies investigating the sequential expression regulation and role of XBP1s and XBP1u will contribute to a better understanding of the mechanism underlying myoblast differentiation, especially in the initial stage of differentiation.

## Conclusion

**FIGURE 6:**
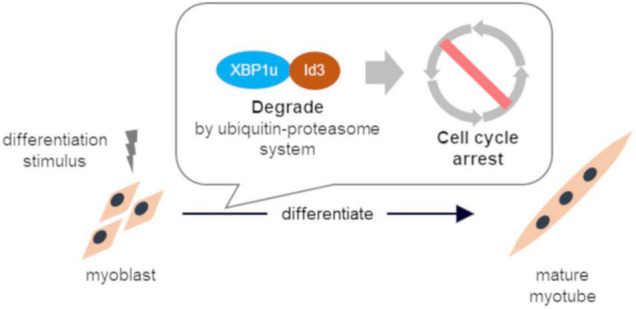
Schematic illustration of conclusion. XBP1u physically bound to ID3 accelerated proteasomal degradation of Id3, a novel regulator of the CDK inhibitor. XBP1u regulated cell cycle exit after differentiation induction *via* interactions with Id3 and promoted myogenic differentiation.

These results indicate that XBP1u regulates cell cycle exit after differentiation induction *via* interactions with Id3 in C2C12 cells (Figure 6). Moreover, this XBP1u-Id3 interaction is necessary for C2C12 myoblast differentiation. To the best of our knowledge, this is the report of the involvement of XBP1u in myoblast differentiation. However, the functions of XBP1u remain largely unknown, although it has been suggested that XBP1u may acts as a “regulator” of myoblast differentiation under various physiological conditions.

## Data Availability Statement

The original contributions presented in the study are included in the article/Supplementary Material, further inquiries can be directed to the corresponding author/s.

## Author Contributions

SH and SY contributed to the experimental design. SH, SS, SK, and YT conducted the experiments and analyzed the data. SH and YT drafted the manuscript. SY supervised the project and corrected the manuscript. All authors contributed to the article and approved the submitted version.

## Conflict of Interest

The authors declare that the research was conducted in the absence of any commercial or financial relationships that could be construed as a potential conflict of interest.

## Publisher’s Note

All claims expressed in this article are solely those of the authors and do not necessarily represent those of their affiliated organizations, or those of the publisher, the editors and the reviewers. Any product that may be evaluated in this article, or claim that may be made by its manufacturer, is not guaranteed or endorsed by the publisher.

## Footnotes

1
https://imagej.nih.gov/ij/

## Supplementary Material

Supplementary Figure 1 **(A)** EdU (green) and DAPI (blue) staining of undifferentiated XBP1 KD or mock cells (left). Quantification of the percentage of EdU-positive nuclei. The results are presented as the mean ± SEM of three independent experiments. Student’s *t* test. ^***^*p* < 0.001 vs. the control group. **(B)** Cell growth curves of XBP1 KD and mock cells. Results are means + SEM for three independent determinations. Student’s *t* test. **p* < 0.05, ^**^*p* < 0.01, and ^***^*p* < 0.001 vs. the control group.
