# Supplementary figures and images for: XBP1u Is Involved in C2C12 Myoblast Differentiation via Accelerated Proteasomal Degradation of Id3
Source: Front Physiol. 2022 Jan 27;13:796190. doi: 10.3389/fphys.2022.796190 (PMC8829448; doi:10.3389/fphys.2022.796190)

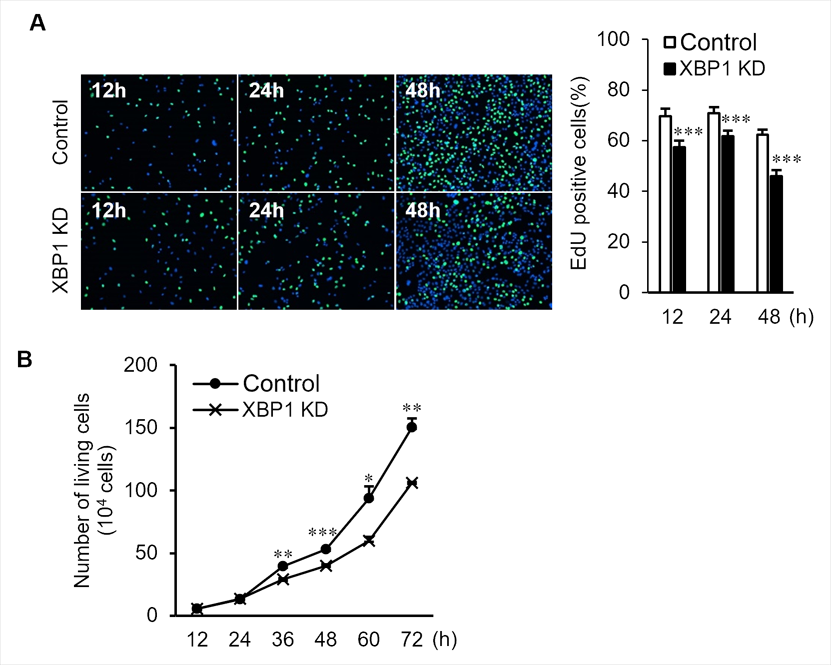

Supplement: Supplementary Figure 1 — (A) EdU (green) and DAPI (blue) staining of undifferentiated XBP1 KD or mock cells (left). Quantification of the percentage of EdU-positive nuclei. The results are presented as the mean ± SEM of three independent experiments. Student’s t test. ***p < 0.001 vs. the control group. (B) Cell growth curves of XBP1 KD and mock cells. Results are means + SEM for three independent determinations. Student’s t test. *p < 0.05, **p < 0.01, and ***p < 0.001 vs. the control group. [file Image_1.TIF]
